# Observation of climatic parameters and plant phenology in the international phenological garden of Klaipėda University Botanic Garden, Lithuania

**DOI:** 10.1007/s00484-025-03067-3

**Published:** 2026-02-02

**Authors:** Asta Klimienė, Ramutis Klimas, Renata Pilkaitytė

**Affiliations:** 1 Klaipėda University Botanic garden, H. Manto str. 84, Klaipėda, 92294 Lithuania; 2 Klaipėda University Marine Research Institute, Universiteto av. 17, Klaipėda, 92294 Lithuania

**Keywords:** Precipitation, Air temperature, Plant phenological data, International phenological garden

## Abstract

The phenology of plants varies greatly over broad geographic gradients, according to climate zone and vegetation type. Phenological records were collected from 2007 to 2024 at KUBG, which is one of the 89 gardens belonging to the International Phenological Gardens (IPG No. 151). The garden is located in Western Lithuania, close to the Baltic Sea coastline (about 3.5 km) (55°42′40″N 21°7′50″E). For the analysis, only 5 species were chosen. The average annual air temperature in Klaipėda is 7.9 °C. The coldest period is January-February, where the average air temperature is -1.0 °C. The warmest period occurs in July-August (aver. 18.2 °C). The autumn temperature of the last season had the most influence: the strong correlation was with leaf unfolding of all trees, as well as strong or moderate correlation with the beginning and full flowering of *Salix viminalis* and *Syringa ×chinensis.* Only the precipitation of last autumn and precipitation of January-February had a statistically significant influence on spring phenophases. *Salix viminalis* had the longest vegetation period (224 days) while the *Sambucus nigra* had the shortest one − 187 days. Precipitation during January-February had a strong or moderate positive correlation with the leaf unfolding of all examined trees, as well as a moderate positive effect for beginning and full flowering of *Syringa chinensis*. The summer and autumn temperature had a negative relation for both vegetation periods. The strongest correlation appeared between summer temperature and vegetation period of *Corylus avellana* and *Sambucus nigra*. These findings highlight the importance of long-term phenological monitoring as a sensitive indicator of climatic variability and as a tool for ecosystem management and climate adaptation strategies.

## Introduction

Phenology is a field of ecological science that studies the timing of periodic biological events in relation to climatic variations, which studies the annual rhythm of the biological development of plants and, the relationship with climatic variations is recognized as a global issue that needs to be investigated at the local level. The strong relationship between air temperature and plant development in the northern hemisphere makes phenological observations a sensitive indicator (Schnelle and Volkert 1974; Chmielewski and Rötzer 2001; Chmielewski et al. 2013; Piao et al. 2019). Although the majority of phenological studies have been conducted in the Northern Hemisphere, research from the Southern Hemisphere is essential to provide a global perspective. To get a more comprehensive view of the global impacts of phenological changes, we need to first focus on research in the global South. Transdisciplinary research needs to become the norm, both to aid progress towards CBD targets and to improve our understanding of the ramifications of these responses to global climate change (Hickinbotham et al. 2025). Phenology varies greatly over broad geographic gradients, according to climate zone and vegetation type, with substantial interannual variability in the timing and duration of the growing season related to the interannual climatic variations. Phenology also varies within populations, and the phenology of individual plants plays a key role in the determination of how ecosystems are structured and how they function (Cleland et al. 2007). According to the authors, the average global temperature has increased by 0.2 °C per decade over the past thirty years. This means, the climate warming process has an influence on the sequence of biological processes (IPCC 2021; Chapin et al. 2008; Hughes 2000; Lesica and Kittelson 2010; Woods et al. 2022).

For phenological observations, countries and regions around the world participate in various networks. One of the longest-running phenological networks is in Europe, where monitoring by the German Weather Service (DWD) began in 1922. Later, observations became part of the International Phenological Garden (IPG) network, which was established in 1959 (Schnelle and Volkert 1974; Chmielewski et al. 2013; http://ipg.hu-berlin.de). The first project PEP725, comprises observations of 139 plants and 33 development phases, late national phenological networks and the International Phenological Gardens (IPGs; http://ipg.hu-berlin.de; Chmielewski and Rötzer 2001). A major goal of phenology is to understand the effects of climate on plant development. The usual phenological network established within a region may elucidate local patterns to some extent, but the information is not precise enough to evaluate research hypotheses. Variations in plant development may arise from hereditary factors, as well as from location ones (Schnelle and Volkert 1974).

Different plant species have evolved different life strategies based on different trade – offs, between survival and capacity adaptations, and consequently different species’ phenological responses are also expected (Downs and Borthwick 1956; Murray et al. 1989; Heide 1993, 2008).

Therefore, studying these differences and their implications is particularly important for evaluating the impacts of climate change at the ecosystem level. The aim of this work was to identify the climatic factors that influence the phenology of different plant species. More broadly, this research seeks to document and analyze phenological patterns, which are essential for understanding periodic biological events in plants and their relationships with seasonal and climatic variations. In addition, the study contributes to understanding how living organisms respond to environmental and climatic changes, thereby providing valuable insights for advancing scientific knowledge, supporting conservation efforts, and improving natural resource management.

## Materials and methods

Phenological records were provided by the International Phenological Gardens (IPG) database, which has been the Physical Geography/Landscape Ecology and Sustainable Ecosystem Development KU Eichstätt-Ingolstadt (https://ipg.ku.de*)* since 2023. The International Phenological Gardens (IPG) conduct large-scale, standardised phenological observations. Therefore, all IPGs are situated in similar surroundings. To eliminate heritable variability, cloned species of all trees and shrubs are planted in the IPGs with a unique ID number that is the same in all gardens.

### Study site

Phenological records were collected from 2007 to 2024 at Klaipeda university botanical garden (KUBG), which is one of the 89 gardens belonging to the International Phenological Gardens (IPG No. 151). The garden is situated in western Lithuania, approximately 3.5 km from the Baltic Sea coast (55°44’58.428”N, 21°8’17.315”E) - altitude − 9 m.

The garden is situated at the riverbank on a plain surface with meadows and some trees. The climate zoning is transitional between the mild maritime climate of Western Europe and the continental climate of Eastern Europe, dominated by westerly air masses coming from the Atlantic Ocean (Galvonaite et al., 2013). The distance between KU Botanic garden and Klaipėda metrology station (LHMS) is about 3 km, the micro-locations are influenced by the sea and surrounded by trees, therefore the data is quite accurate. Therefore, during winters, the air temperature is a few degrees below freezing, while summers are mild to pleasantly warm. The average annual air temperature in Klaipėda is 7.9 °C. The coldest period is January-February, when the average air temperature is −1.0 °C (min − 26.0 °C). The warmest period occurs in July-August (average 18.2 °C, max 36.6 °C (LHMS 2025), Fig. 1A). Over the last half century, the average annual air temperature in Klaipeda has increased by 1.2 °C (Dailidienė et al. 2023). Meanwhile, the number of days when daily air temperatures are negative decreased by 0.71 days per year, or 22.7 days in 32 years (Fig. 2, data were taken from LHMS (2025).

**Fig. 1:**
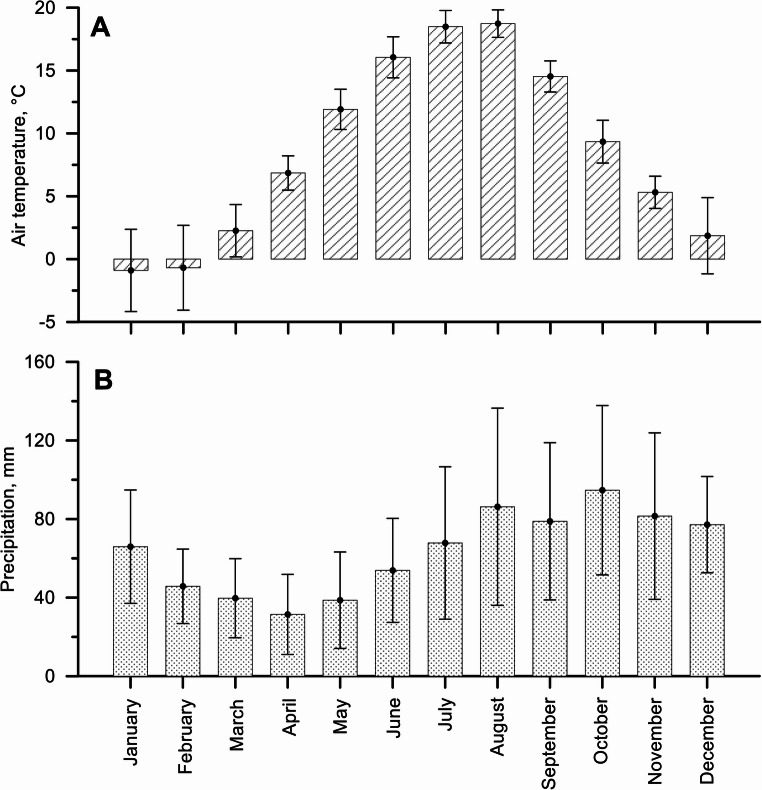
Long term (y. 1991–2024) data at Klaipėda meteorological station: **A** – monthly average (± SD) of air temperature, **B** – monthly sum (± SD) of precipitation. Daily data taken from LHMS (2025)

**Fig. 2:**
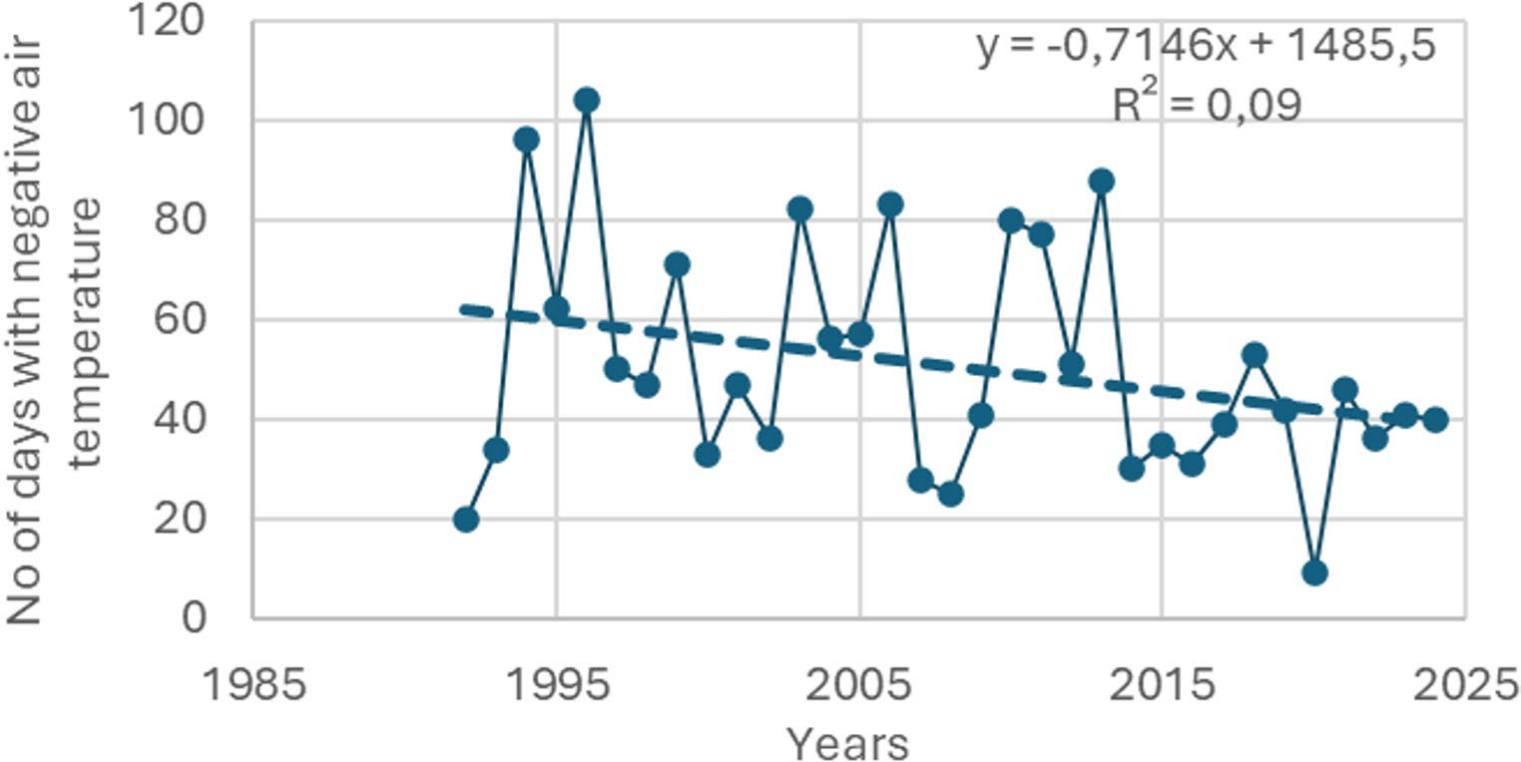
The number of days with average negative daily air temperatures. Data from LHMS (2025)

The long-term average annual precipitation on the coastal area is 762 mm per year (1991–2024, LHMS 2025). The driest period is in the spring – April (monthly mean/min/max – 31/2.5/73 mm), the wettest period is in October (monthly mean/min/max – 95/8.9/204 mm) (Fig. 1B).

### Phenological observations

KUBG has been a part of the IPG network since 2005, while plant observation started in 2006. In the first step, 13 cloned plants were obtained from Humboldt University in Berlin (Germany).

In 2018, there were 19 species growing and being observed. However, for the analysis, only 5 species were chosen; others were disregarded because the plants did not bloom due to their youth. The selected indicator species were: *Corylus avellana*, *Salix ×smithiana*, *Salix viminalis*, *Sambucus nigra*, and *Syringa ×chinensis* ‛Red Rothomagensis’ (Table 1). All these species are common in the Northern Hemisphere and in Lithuania too, except the last two species (*S. nigra* and *S. chinensis*), which are not native to Lithuania, and their vegetation period begins in late spring or early summer. The other three species are native, and their vegetation period starts in early spring. The data of all observed plants were taken from the IPG page (IPGs; https://ipg.ku.de) (Table 1). An 18-year data series (2007–2024) was used for the analysis. In this study, the 5 vegetation phenophases were observed: leaf unfolding (UL), beginning of flowering (BF), full flowering (FF), autumn leaf colouring (CL), and leaf fall (FL), as well as the two plant vegetation periods, one from the beginning of leaf unfolding up to leaf colouring (UL-CL), and second, from beginning of leaf unfolding up to leaf fall (UL-FL) because for certain species in our study, leaf colouring is not prominent, whereas leaf fall is clearly observable.

**Table 1:** List of plants investigated in Klaipėda UBG phenological garden

No.	Identify No.	Species of the plant, origin	Locality	Taxonomic family
1	324	*Salix* ×*smithiana* Willd. (Germany) = *S. smithiana* The basket willow grows in northern continental Europe and in North Asia. It is cultivated to produce wickerwork like baskets of the extremely long rods. In KUBG IPG height 8.2 m, width – 6 m.	Local	*Salicaceae* Mirb.
2	326	*Salix viminalis* L. (Germany) = *S. viminalis* The basket willow grows in northern continental Europe and in North Asia (native to Europe, Western Asia, and the Himalayas). It is cultivated to produce wickerwork like baskets of the extremely long rods. In KUBG IPG height – 5.2 m, width – 9 m.	Local	*Salicaceae* Mirb.
3	331	*Sambucus nigra* L. (Germany) = *S. nigra* The common elder is one of the most common shrub species in Central Europe. It has been introduced to parts of most other continents of the world. Both the flowers and the berries have a long tradition of culinary use. In KUBG IPG height 4 m, width – 3 m.	Introduced	*Sambucaceae* Batsch ex Borkh.
4	411	*Corylus avellana* L. = *C. avellana* The common hazel is widespread in large parts of Europe, Asia Minor, and the Caucasus. It is known for its edible hazel nuts. In KUBD IPG height 6.5 m, width – 4.5 m.	Local	*Betulaceae* Gray
5	431	*Syringa* ×*chinensis* ‛Red Rothomagensis’ = *S. chinensis* *Syringa* is native to woodland and scrub from southeastern Europe to eastern Asia, and widely and commonly cultivated in temperate areas elsewhere. *Syringa* ×*chinensis* was a result of hybridisation of several species in 1770 in Rouen (France). In KUBG IPG height 3 m, width – 2.5 m.	Introduced	*Oleaceae* Hoffmanns et Link.

### Data analysis

Firstly, statistical correlations were tested between monthly and interseasonal variations of temperature (monthly average) and precipitation (monthly sum) as abiotic factors, and different phenophases. The best correlations appeared when seasonal data were applied. Therefore, the latest were used for the redundancy analysis (RDA). Both correlation and RDA were applied to test the relationships between the abiotic factors (seasonal average of air temperature, number of days with average negative daily temperature, and sum of precipitation) used as explanatory variables and trees’ phenophases (UL, BF, FF, CL, FL, UL-CL, and UL-FL) as response variables, using Brodgar (2000) and R (3.3.3) packages. Brodgar generated RDA biplots that were interpreted based on the directions and lengths of explanatory factor lines and response variable lines (Zuur et al. 2007). For the spring phenology, the climatic data (summer, autumn) of previous years were used; therefore, the RDA was applied separately for spring and autumn plant phenology.

## Results

**Fig. 3:**
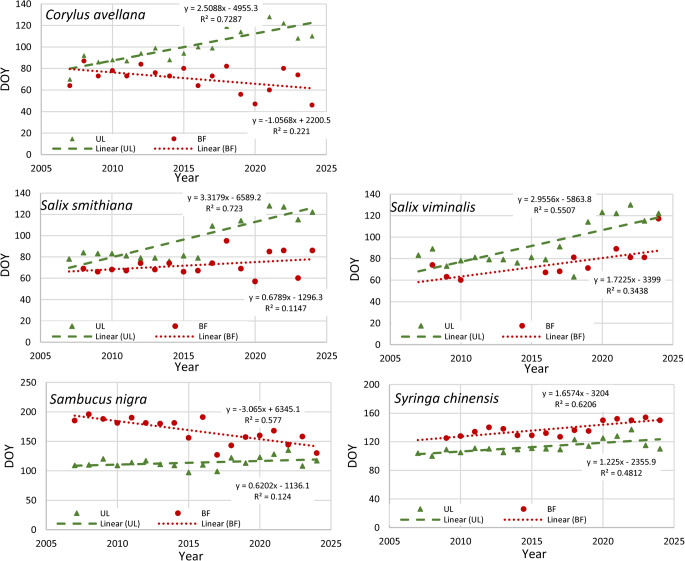
Changes in beginning of leaf unfolding (UL, green triangles and linear regression line) and the beginning of flowering (BF, red dots and linear regression line), shown in days starting from January 1 (DOY)

**Fig. 4:**
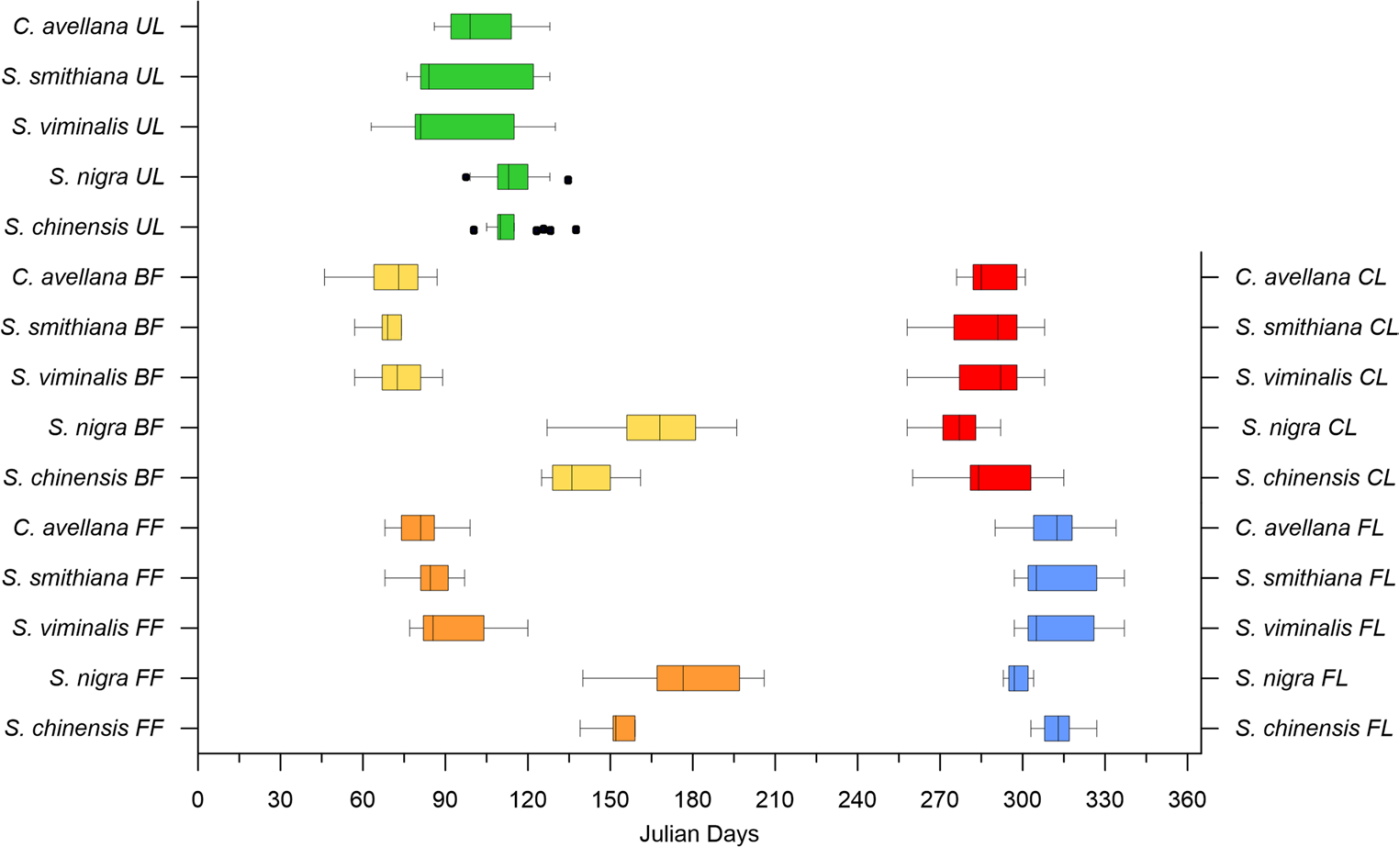
Plant phenophases (Box plots min, max, median, and 25% and 75% quartiles). UL – beginning of leaf unfolding, BF – beginning of flowering, FF – full flowering, CL – beginning of leaf colouring, FL – beginning of leaf fall

A comparison of the phenophases of all studied plants revealed several distinctive characteristics. Vegetation (UL) of early species started on average after 93 (± 21), 98 (± 21), and 101 (± 16) days (in days from 1 st January) for *S. viminalis*, *S. smithiana*, and *C. avellana*, respectively. For these three species, beginning of flowering (BF) and full flowering (FF) phenophases started earlier than leaf unfolding (UL) (Figs. 3 and 4). The non-local species *S. nigra* and *S. chinensis* started their growing season later, around April 22nd −23rd (day 114 ± 9) on average (Figs. 3 and 4).

All studied plants, except *S. nigra*, showed a delay in the leaf unfolding during the last 18 years. The most pronounced and significant delay was observed for the *C. avellana*,* S. smithiana* and *S. viminalis* (Fig. 3). However, the beginning of flowering for the *S. nigra* tended to be earlier compared to the 18 previous years (Fig. 3). The other plants didn’t show any significant changes. For all tree species studied, full flowering (FF) began on average 11–15 days after the beginning of flowering (Fig. 4).

**Fig. 5:**
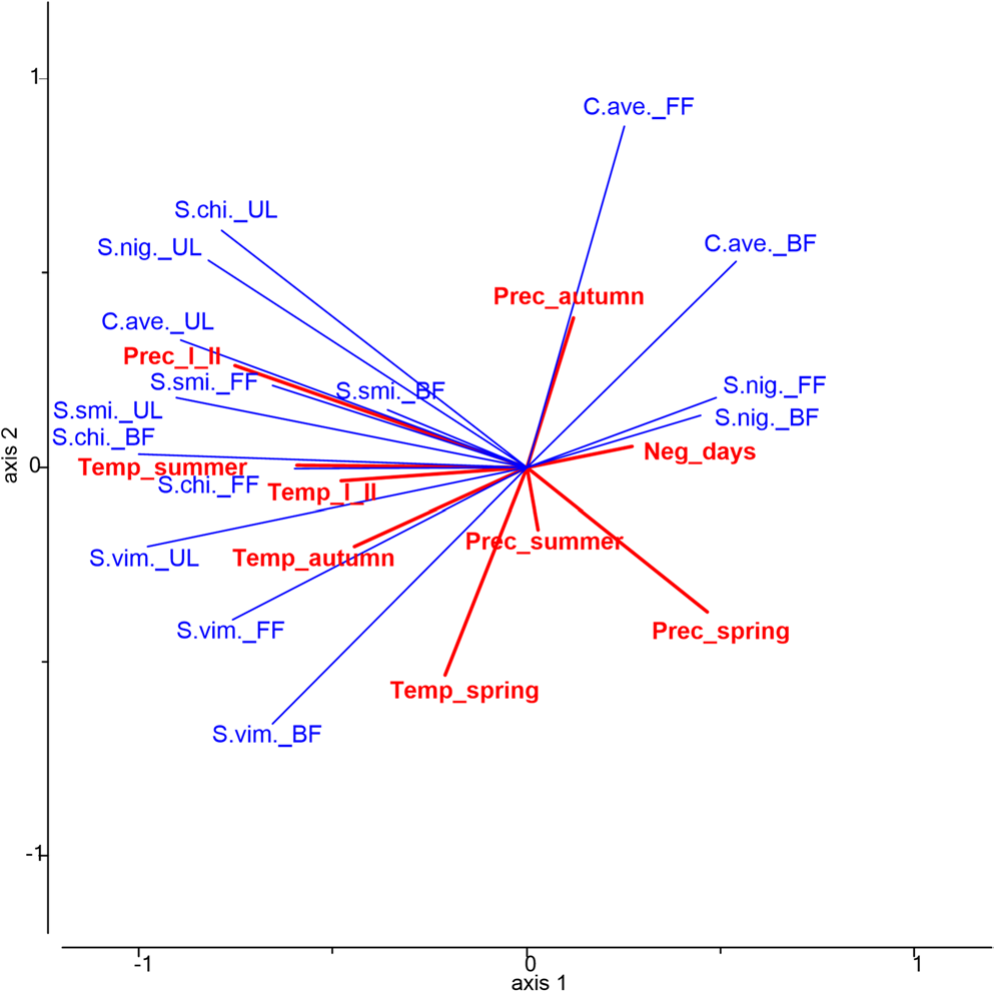
Influence of the monthly sum of precipitation (Prec_) and the monthly average of temperature (Temp_) of different seasons, and the sum of days with negative average air temperature (Neg_days) of the previous season on trees’ spring phenology. (I_II – January and February, C.ave. – *C. avellana*, S.smi. – *S. smithiana*, S.vim. – *S. viminalis*, S.nig. – *S. nigra*, S.chi. – *S. chinensis*, UL – beginning of leaf unfolding, BF – beginning of flowering, FF – full flowering)

Based on the RDA analysis, the environmental variables contributed to 56% of the variability observed in the spring phenological characteristics, with two axes capturing 74% of the total variation (Fig. 5). Only precipitation in January-February (Prec_I_II) had the statistically significant influence on spring phenophases, it explained 33% (*p* = 0.003) of the variation.

Precipitation from last autumn explained 11% of the variation and showed a moderate positive correlation with full flowering of *C. avelana*, while exhibiting a moderate negative correlation with leaf unfolding of *S. viminalis*. Precipitation during January-February had a strong or moderate positive correlation with leaf unfolding of all examined trees, as well as a moderate positive effect on the beginning and full flowering of *S. chinensis*. However, precipitation during the spring (explained 16% of variation) had a negative correlation with leaf unfolding of some examined trees: *C. avelana*,* S. smithiana* and *S. chinensis*.

No single season’s temperature could significantly explain the variation in spring phenology timing. Nevertheless, variations in January–February temperatures explained 18% of the variability observed in spring phenophases, while autumn temperatures accounted for 15% of the observed variation. The most influential factor was the autumn temperature of the last season: strong or moderate correlations were with leaf unfolding of all trees, as well as a moderate correlation with beginning and full flowering of *C. avelana*. The temperature of January-February had a moderate positive correlation with the leaf unfolding of *S. viminalis* and with the flowering phenology of *S. chinensis*.

The sum of days with negative average air temperature (Fig. 5) of the previous season explained only 7% of the variation and was closely related to the *S. nigra* full flowering phases but was not statistically significant.

**Fig. 6:**
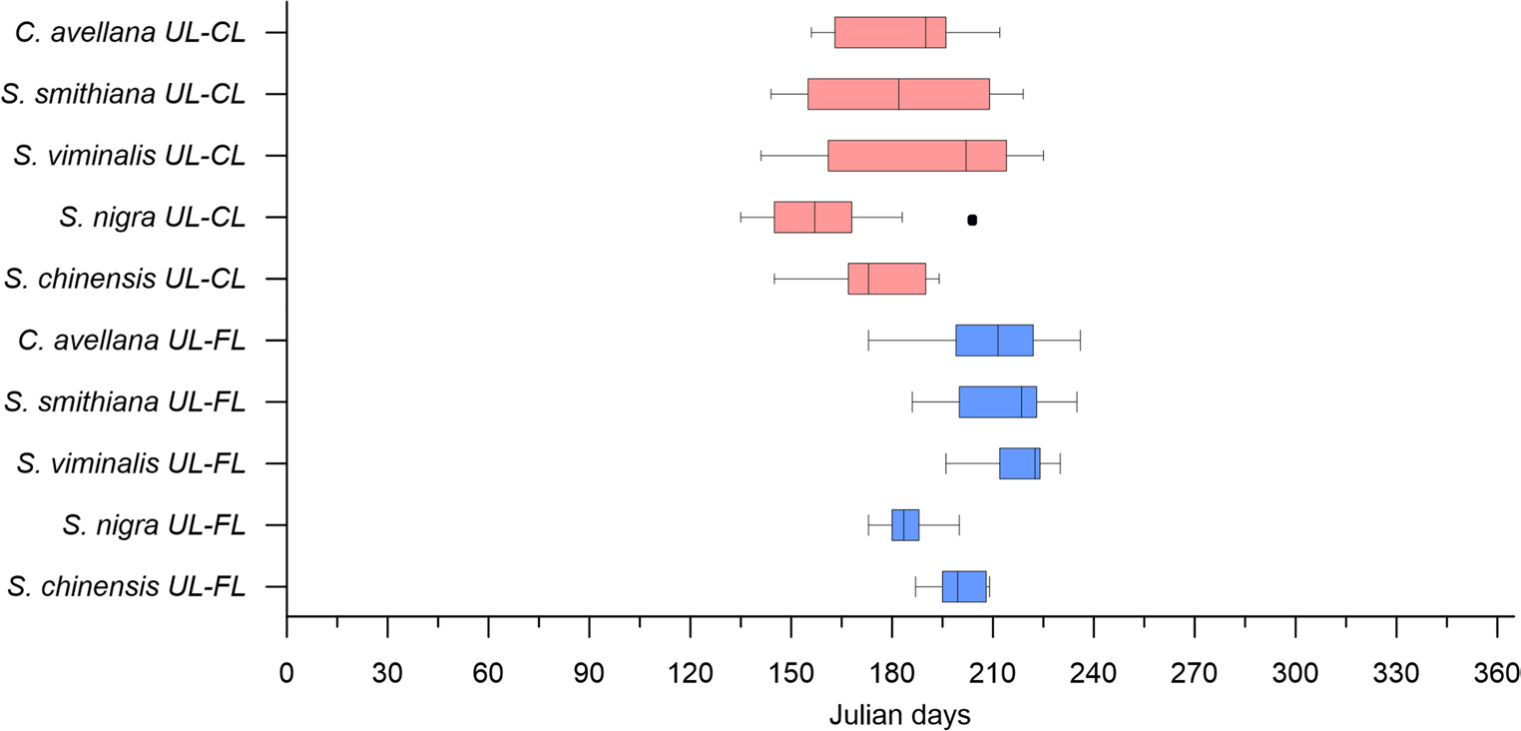
Duration of the plant vegetation periods from the beginning of leaf unfolding till leaf colouring (UL-CL, top bars) and till leaf fall (UL-FL, bottom bars)

In the examined plants, leaf colouring primarily began in October (Fig. 6). Throughout the observation period, *S. smithiana* began leaf colouration the earliest, on August 14, 2007. In contrast, *Sambucus nigra* demonstrated the earliest average onset of both leaf colouration and leaf fall among the remaining species, occurring on October 1 st and 22nd, respectively. The other species showed a delay in these phenophases of approximately one to two weeks.

**Fig. 7:**
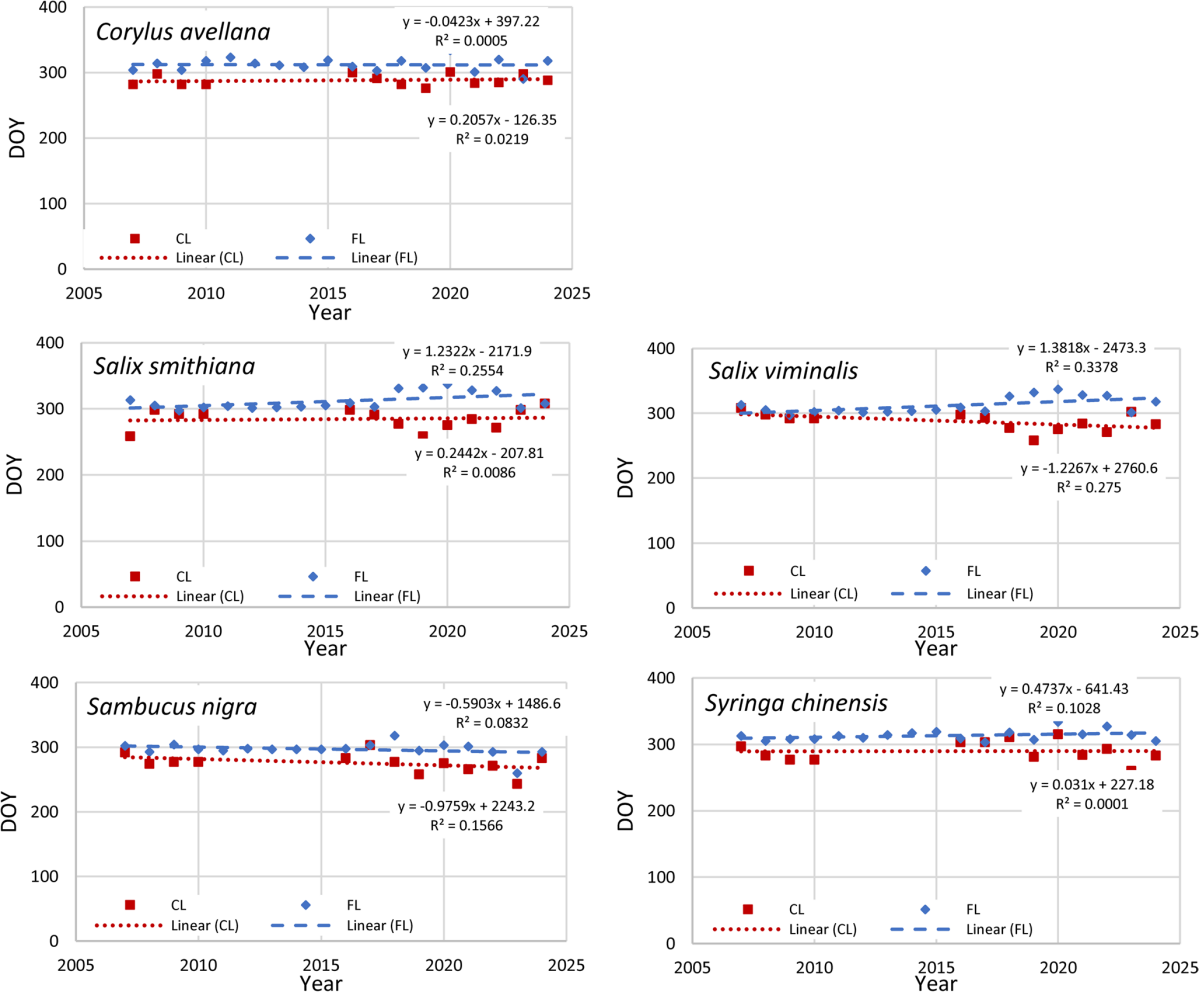
Changes in beginning of leaf colouring (CL, red squares and linear regression lines) and the number of days till leaf fall (FL, blue rhombus and linear regression lines), shown in days starting from January 1 (DOY)

Autumn phenophases were stable for all examined plants over the last 18 years, except *Salix* species. The leaf colouring tends to appear slightly earlier of *S. viminalis*, while leaf fall begins slightly later of both *Salix* species over the last 18 years (Fig. 7).

The vegetation period from the beginning of leaf unfolding till leaf colouring (UL-CL) appeared much more varied compared to the vegetation period from UL until FL (UL-FL). Nevertheless, the longest vegetation period on average was *S. viminalis* 219 ± 17 days (UL-FL) and 188 ± 32 days (UL-CL), while the *S. nigra* had the shortest one – 183 ± 13 days (UL-FL) and 160 ± 20 days (UL-CL) (Fig. 6).

**Fig. 8:**
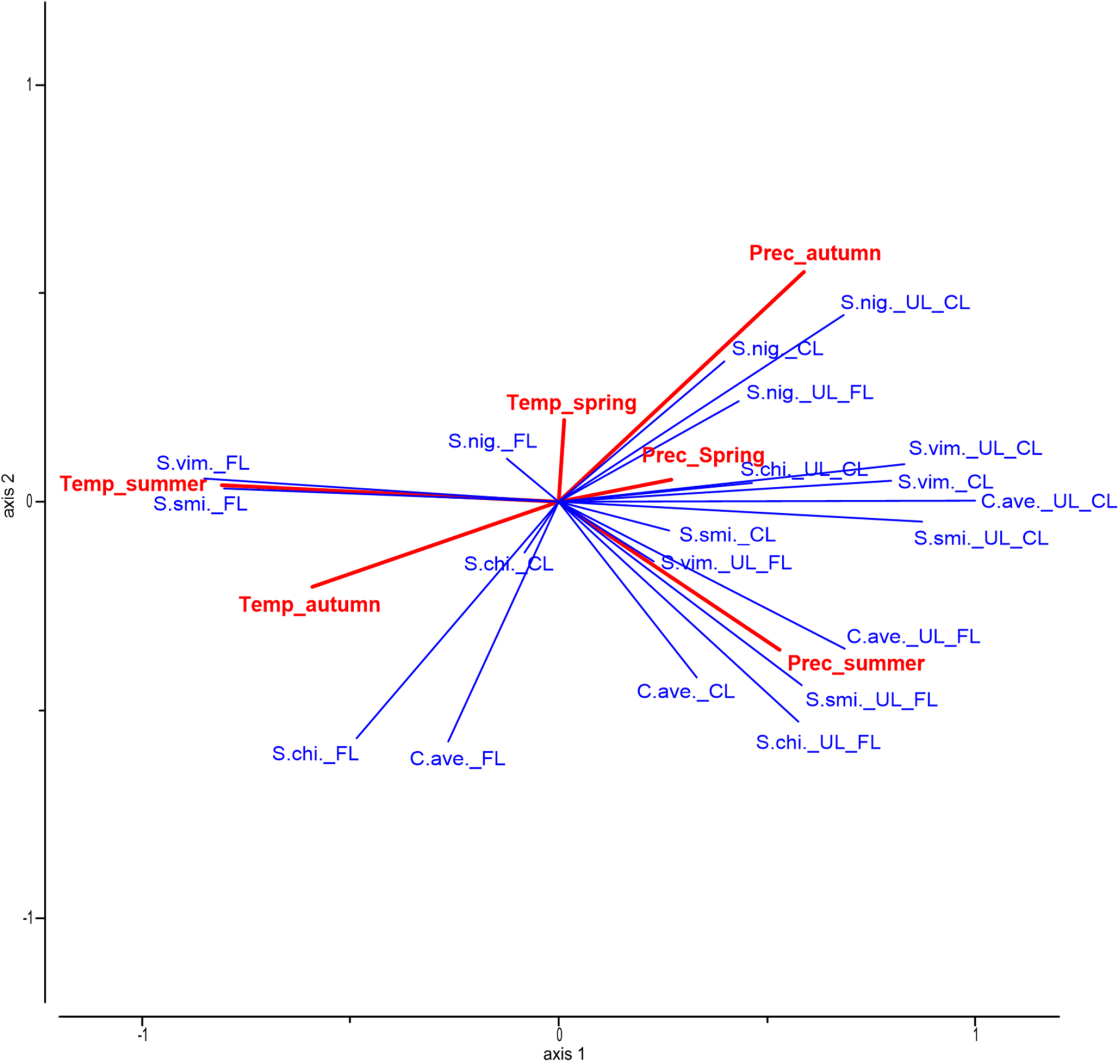
Influence of the season sum of precipitation (Prec_) and the monthly average of temperature (Temp_) of different seasons on to trees’ autumn phenology. (C.ave. – *C. avellana*, S.smi. – *S. smithiana*, S.vim. – *S. viminalis*, S.nig. – *S. nigra*, S.chi. – *S. chinensis*, CL – beginning of leaf colouring, FL – beginning of leaf fall, UL_CL – vegetation from the beginning of the unfolding leaves till leaf colouring, and UL_FL – vegetation from the beginning of the unfolding leaves till leaf fall)

According to the RDA analysis, the environmental factors accounted for 45% of the variance in the autumnal phenophases and vegetation period duration, while two axes explained 80% of the variation (Fig. 8). The different plant species reacted differently to the climatic factors. The most significant influence on autumnal phenological phases was summer air temperature, explaining 43% (*p* = 0.001) of the variability. Air temperature of autumn explained 26%, while precipitation during summer and autumn accounted for 23% and 28% of the variability respectively. However, the influences of these parameters were not statistically significant.

The summer temperature had a moderate positive effect on the beginning of leaf fall of both *Salix* species, as well as weak (for *S. smithiana*,* S. nigra*, and *C. avellana*) or moderate (for *S. viminalis*) negative correlation for the beginning of leaf colouration, i.e. the higher the temperature in the summer the earlier leaf colouration starts. However, the autumnal higher temperature had a moderate positive effect on the beginning of leaf fall of both *Salix* species and was weak to *S. chinensis*, i.e. the leaf fall started later.

Higher amounts of precipitation in the summer and autumn had weak positive or no influence on the beginning of leaf colouration; however, these factors stimulated earlier leaf fall. The correlation of precipitation during the summer was weak with *S. nigra* and both Salix species, while precipitation during autumn had a moderate correlation with both *Salix* species and *S. chinensis* and was weak with *C. avellana*.

The summer and autumn temperatures had a negative correlation for both vegetation periods (UL-FL and UL-CL). The strongest correlation appeared between summer temperature and UL-CL vegetation period of *C. avellana* and *S. nigra*. For other species, the correlation was moderate.

However, precipitation had the opposite effect. More precipitation during the summer extended the vegetation season for *C. avellana* (both for UL-FL and UL-CL), *S. smithiana* (UL-FL), and *S. chinensis* (UL-FL), while precipitation in autumn prolonged the vegetation season for *S. nigra* (UL-CL, UL-FL) and *C. avellana* (UL-CL).

## Discussion

Phenological observations are one of the most important (and sometimes the only) sources of information on the physiological condition of plants and their reactions to external forcing (Sparks and Menzel 2002; Menzel et al. 2006; Klimienė, et al., 2016). Climate is a dynamic system that changes gradually over time. From 1961 to 2020, the average annual air temperature along the Baltic Sea coast in Lithuania rose by 1.2 °C (Dailidienė et al. 2023). The number of days with average negative daily air temperatures has decreased by 10 days over the past 30 years (Fig. 2). Although the annual amount of precipitation in Klaipeda changed slightly, intense precipitation, when 20 mm or more precipitation falls per day, increased (LHMS 2025). Plants tend to adapt to changes by adjusting their phenology. Long-term observations and systematic data collection are essential for monitoring climate change, conducting thorough analysis, and drawing realistic conclusions.

Climate analyses indicate a strong warming during the winter months across Europe (EEA 2004). Over the past 40 years, there has also been a significant increase in summer air temperatures. The smallest temperature increase has been observed in autumn; even, a slight decrease (− 0.1 °C) can be seen in October (Dailidienė et al. 2023). This aligns with the observations of seasonal temperature patterns, where winter shows a slight but consistent warming trend in Klaipėda region as well (Dailidienė et al. 2023; LHMS 2025). This may explain why our observations indicated a stronger impact of climate change on spring phenophases (Fig. 3) than on autumnal ones (Fig. 7). The beginning of phenological spring in Lithuania is related to the start of the vegetation of *C. avellana*, which is indicated differently by different authors, for example, Klimienė et al. (2016) indicated *C. avellana* beginning of blooming in North Lithuania on 4th of April on average, Romanovskaja et al. (2012) specified March 27th, while our the latest observation data showed that flowering has begun on March 10th on average.

In Eastern European countries, the vegetation of *C. avellana* is more distinct and exhibits a stronger dependence on climatic factors. Comparison of the onset of flowering of local *C. avellana* at KUBG IPG indicates that during 2007–2024, it occurred 14 days earlier than in the reference period 1961–2010. Kalvane et al. (2009) had evaluated that in the Baltic countries, Latvia and Lithuania, during the observation period of 1971–2000, *Corylu*s vegetation started earlier in locations closer to the Baltic Sea. Data also showed that the plants with the earliest spring leaf unfolding were very sensitive to climate change. Ahas et al. (2002) studies show that throughout the 1951–1998 spring phenological phases of *C. avellana* began earlier in Western Europe and the Baltic Sea regions. Thus, this species is probably one of the most climate-affected species in terms of phenology. Data from the PEP725 phenological database indicate a shift toward earlier flowering and delayed leaf unfolding in *C. avellana* across Europe (http://www.pep725.eu), aligning with our 18‑year observation that flowers appear earlier while leaves are unfolding later despite warmer temperatures. Other observed trees (*Salix*,* Syringa*) also showed similar delays in leaf unfolding (Fig. 3). Most probably, it was not the increase in temperature that affected it, but the tendency of the air temperature transition from 0 °C to a higher temperature to be delayed, i.e., the delay in the beginning of spring (Galvonaitė et al. 2007). Meanwhile, it has been observed that higher summer temperatures lead to faster leaf colouration and shorter vegetation UL-CL period, but later leaf fall. Despite the obvious influence of temperature, its impact was statistically significant only on autumnal phenophases.

So phenological data also allow researchers to identify critical climate change indicators, such as the effect of winter warming on the onset of spring phases or the impact of precipitation patterns on leaf development. Systematic long-term observations enable assessment of interseasonal climate variability, understanding of ecosystem adaptation mechanisms and development of predictive models for biological processes under changing climatic conditions.

Hydrological data suggest divergent seasonal precipitation trends across Europe: winter precipitation has increased in many regions, whereas summer and autumn precipitation have shown a decreasing or stable trend (Kovats et al., 2014; IPCC 2021). This applies to Klaipeda as well, with rising winter rainfall and declining autumnal and summer precipitation (Galvonaitė et al. 2007; LHMS 2025). Although those changes are small – a few or a few tens of millimetres - higher precipitation in the winter months resulted in later leaf unfolding, and this effect was statistically significant (Fig. 8). Precipitation in Western Lithuania is the lowest in the spring months (Fig. 1B), and a trend was observed – more precipitation during these months promoted faster leaf spreading.

Comprehensive understanding of plant responses to climate change and the development of robust predictive models require consistent, long-term studies. Although such studies are gradually emerging, they remain largely isolated and country-specific, underscoring the need for systematic analysis and aggregation of existing datasets. Because phenological events of plants across biogeographical regions are particularly poorly documented (Templ et al. 2017).

## Conclusions

This study found that the mean autumn temperature of the previous year was the primary factor influencing spring phenological events, particularly leaf unfolding across all studied tree species. Strong to moderate correlations were also observed between temperature and the flowering phenology of *Salix viminalis* and *Syringa × chinensis*. *Corylus avellana* and *Sambucus nigra* showed earlier flowering compared to the 15-year average, while no significant phenological changes were noted in other species.

Environmental factors explained a large portion of the variation in spring phenology. Precipitation in the preceding autumn and in January–February had the strongest impact. Warmer temperatures during the previous summer, autumn, and winter led to earlier flowering in *Corylus avellana*. Vegetation periods differed by species, with *Salix viminalis* having the longest and *Sambucus nigra* the shortest.

Precipitation effects varied by season and species, with notable positive and negative correlations depending on timing and phenophase. These findings underscore the importance of phenological monitoring for understanding plant responses to climate variability and for informing long-term environmental assessments, biodiversity conservation, and climate policy development.

These insights provide a scientific basis for regional climate adaptation planning and biodiversity monitoring initiatives in coastal Lithuania.

## Data Availability

The datasets analysed during the current study are available from the corresponding author on reasonable request.

## Code Availability

(software application or custom code) Not applicable.

## References

[CR1] Ahas R, Aasa A, Menzel A, Fedotova VG, Scheifinger H (2002) Changes in European spring phenology. Int J Climatol 22:1727–1738

[CR2] Brodgar (2000) Software package for multivariate analysis and multivariate time series analysis, version 2.7.5. Highland Statistics Ltd.

[CR3] Chapin FS, Matson PA, Vitousek PM (2008) *Principles of terrestrial ecosystem ecology* (2nd ed.). Springer. 10.1007/978-1-4020-5599-5

[CR4] Chmielewski FM, Rötzer T (2001) Response of tree phenology to climate change across Europe. Agric For Meteorol 108:101–112

[CR5] Chmielewski FM, Heider S, Moryson S, Bruns E (2013) International Phenological Observation Networks: the concept of IPG and GPM. In: Schwartz DM (ed) Phenology: An Integrative Environmental Science. Springer, Dordrecht Heidelberg, New York, pp 137–153

[CR6] Cleland EE, Chuine I, Menzel A, Mooney HA, Schwartz MD (2007) Shifting plant phenology in response to global change. Trends Ecol Evol 22:357–365 17478009 10.1016/j.tree.2007.04.003

[CR7] Dailidienė I, Servaitė I, Dailidė R, Vasiliauskienė E, Rapolienė L, Povilanskas R, Valiukas D (2023) Increasing trends of heat waves and tropical nights in coastal regions (the case study of Lithuania seaside cities). Sustainability 2023:14281. 10.3390/su151914281

[CR8] Downs RJ, Borthwick HA (1956) Effects of photoperiod on growth of trees. Bot Gaz 117:310–326

[CR9] EEA (2004) European Environment Agency. Impacts of Europe’s changing climate. An indicator-based assessment. EEA report. No 2/2004

[CR11] Galvonaitė A, Misiūnienė M, Valiukas D, Buitkuvienė MS (2007) Lietuvos Klimatas. VU leidykla, Vilnius. (In Lithuanian)

[CR12] Galvonaitė A, Valiukas D, Kilpys J, Kitrienė Z, Misiūnienė M (2013) Climate. 2013 Atlas of Lithuania. Vilnius. Lithuanian Hydrometeorological Service under the Ministry of Environment; 175p. Available online: http://www.meteo.lt/documents/20181/102884/Klimato+Atlasas+smal.pdf/08c97c20-bd46-4e65-a069-3a0774e4b748 (accessed on 7 February 2023)

[CR13] Heide OM (1993) Dormancy release in beech buds (*Fagus sylvatica*) requires both chilling and long days. Physiol Plant 89:187–191

[CR14] Heide OM (2008) Interaction of photoperiod and temperature in the control of growth and dormancy of *Prunus* species. Sci Hortic 115:309–314

[CR15] Hickinbotham EJ, Ridley FA, Rushton SP, Pattison Z (2025) 30 years of climate related phenological research: themes and trends. Int J Biometeorol 69:1459–1473. 10.1007/s00484-025-02903-w 40353905 10.1007/s00484-025-02903-w PMC12141420

[CR17] Hughes L (2000) Biological consequences of global warming: is the signal already apparent? Trends Ecol Evol 15(2):56–61. 10.1016/S0169-5347(99)01764-4 10652556 10.1016/s0169-5347(99)01764-4

[CR18] IPCC (2021) Climate Change 2021: The Physical Science Basis. Contribution of Working Group I to the Sixth Assessment Report of the Intergovernmental Panel on Climate Change. Cambridge University Press. https://www.ipcc.ch/report/ar6/wg1/

[CR19] Kalvāne G, Romanovskaja D, Briede A, Bakšienė E (2009) Influence of climate change on phenological phases in Latvia and Lithuania. Clim Res 39:209–219

[CR20] Klimienė A, Vainorienė R, Klimas R (2016) Phenological research of climate changes in the North part of Lithuania by the phenological garden of Šiauliai university. Int J Biometeorol 61:293–301 27604576 10.1007/s00484-016-1211-2

[CR21] Kovats RS, Valentini R, Bouwer LM, Georgopoulou E, Jacob D, Martin E, Soussana JF (2014) Europe. Climate Change 2014: Impacts, Adaptation, and Vulnerability. Cambridge University Press, pp 1267–1326

[CR22] Lesica P, Kittelson PM (2010) Precipitation and temperature are associated with advanced flowering phenology in a semi-arid grassland. J Arid Environ 74(8):1013–1017

[CR23] LHMS (2025) Lithuanian Hydrometeorological Service at the Ministry of Environment. http://www.meteo.lt/lt/skn (accessed on 4 June 2025)

[CR24] Menzel A, Sparks TH, Estrella N, Koch E, Aasa A, Ahas R, Alm-Kübler K, Bissolli P, Braslavská O, Briede A, Chmielewski FM, Crepinsek Z, Curnel Y, Dahl Å, Defila C, Donnelly A, Filella Y, Jatczak K, Måge F, Mestre A, Nordli Ø, Peñuelas J, Pirinen P, Remišová V, Scheifinger H, Striz M, Susnik A, van Vliet AJH, Wielgolaski F-E, Zach S, Zust A (2006) European phenological response to climate change matches the warming pattern. Glob Change Biol 12:1969–1976

[CR25] Murray MB, Cannell GR, Smith RI (1989) Date of budburst of fifteen tree species in Britain following climatic warming. J Appl Ecol 26:693–700

[CR26] Piao S, Liu Q, Chen A, Janssens IA, Fu Y, Dai J, Liu L, Lian X, Shen M, Zhu X (2019) Plant phenology and global climate change: current progresses and challenges. Glob Change Biol 25(6):1922–1940 10.1111/gcb.14619 30884039

[CR27] Romanovskaja D, Bakšienė E, Ražukas A, Tripolskaja L (2012) Influence of climate change on the European Hazel (*Corylus avellana* L.) and Norway maple (*Acer platanoides* L.) phenology in Lithuania during the period 1961–2010. Balt for 18:228–236

[CR28] Schnelle F, Volkert E (1974) Phenology and seasonality modelling. Int Phenological Gardens Europe Basic Netw Int Phenological Observations 8:383–387

[CR29] Sparks TH, Menzel A (2002) Observed changes in seasons: an overview. Int J Climatol 22:1715–1725

[CR30] Templ B, Templ M, Filzmoser P, Lehoczky A, Bakšienè E, Fleck S, Gregow H, Hodzic S, Kalvane G, Kubin E, Palm V, Romanovskaja D, Vučetić V, Žust A, Czúcz B (2017) Phenological patterns of flowering across biogeographical regions of Europe. Int J Biometeorol 6(7):1347–1358 10.1007/s00484-017-1312-6 28220255

[CR31] Woods HA, Dillon ME, Pincebourde S (2022) The roles of microclimatic diversity and of behavior in mediating the responses of ectotherms to climate change. J Exp Biol, 2*25*(Suppl_1). 10.1016/j.jtherbio.2014.10.002 26615730

[CR32] Zuur AF, Ieno EN, Smith GM (2007) Analysing Ecological Data. Springer, New York, NY, USA

